# To Cover Pills or Extract of Copaiba with Gelatine

**Published:** 1846-11

**Authors:** 


*To cover Pills or extract of Copaiba with Gelatine.*—This process, invented by M. Garot, is exceedingly easy and practicable, and it is not employed more generally in this country, as it much more effectually disguises the taste and odour, and interferes less with the solution of the medicine, than the method of gilding or silvering usually practised. It is applicable to every substance capable of a pilular consistence; such as balsam, camphor, musk, assafœtida, mercurial and ferruginous preparations, &c. Two hundred pills can be coated with gelatine in an hour, and will be ready for use after the lapse of two hours. The pilular mass so coated remains soft for a much longer time than according to any other plan. The process is as follows:—Fix the pills on long, fine pins; plunge them into thick purified glue placed in a hot-water bath; then remove them by a rotatory motion, and stick the pins in paste spread out on a slab, so that the pills may remain elevated in the air; as soon as fifty are thus treated, rotate them individually in the heat of a taper, to harden the external pellicle, pull out the point of the pin, and the process is complete.—*Ib., from Dublin Hospital Gazette.*

